# Piling it on: Perceived stress and lack of access to resources among US-based LGBTQ+ community members during the COVID-19 pandemic

**DOI:** 10.1371/journal.pone.0271162

**Published:** 2022-07-08

**Authors:** Brooke A. Levandowski, Susan B. Miller, Davy Ran, Eva A. Pressman, Timothy Van der Dye

**Affiliations:** 1 Department of Obstetrics and Gynecology, University of Rochester Medical Center, Rochester, New York, United States of America; 2 Department of Medicine, University of Rochester Medical Center, Rochester, New York, United States of America; University of Perugia: Universita degli Studi di Perugia, ITALY

## Abstract

**Objectives:**

While the LGBTQ+ community has been disproportionally impacted by COVID-19 medical complications, little research has considered non-medical impact.

**Methods:**

We conducted a secondary analyses of USA-based respondents from a global cross-sectional online mixed-methods survey collecting sexual orientation, gender identity, and the perceived stress scale (PSS). Bivariate and multivariate ordinal regression statistics were performed.

**Results:**

Fourteen percent (n = 193,14.2%) identified as LGBTQ+. Variables significantly associated with LGBTQ+ included: COVID testing/treatment affordability, canceled activities, stocking food/medications, quitting job, lost income, and inability to procure groceries/cleaning supplies/medications. Adjusting for Hispanic ethnicity and income, BIPOC LGBTQ+ individuals had twice the odds (OR:2.02;95%CI:1.16–3.53) of moderate compared to low PSS scores, and high compared to moderate PSS scores, compared to white non-LGBTQ+ individuals. Adjusting for Hispanic ethnicity, income, age, and education, deaf LGBTQ+ individuals had twice the odds (OR:2.00;95%CI:1.12–3.61) of moderate compared to low PSS scores, and high compared to moderate PSS scores, compared to hearing non-LGBTQ+ individuals.

**Conclusion:**

The LBGTQ+ community has increased stress due to COVID-19. Public health interventions must mitigate stress in BIPOC and deaf LGBTQ+ communities, addressing their intersectional experiences.

## Introduction

SARS-CoV-2 (COVID -19) emerged in Wuhan, China, in December 2019, and quickly spread worldwide. The first case detected in the United States was in Washington State on January 21, 2020 [1]. At the time of study data collection in May 2020, over 1.8 million people in the US had tested positive for COVID-19 and more than 107,000 people had died; a vaccine had not yet been developed.

At the time of writing in June 2022, about 85 million people in the US have tested positive for COVID-19, more than one million people have died and 221 million people have been fully vaccinated against COVID-19 [2].

The COVID-19 pandemic and early efforts to slow the spread of the virus, including shut downs, caused multitudes of secondary issues, including unprecedented job loss, layoffs, and reduced hours, leading to widespread economic insecurity [3, 4]. Financial loss, the pandemic, and responses to the pandemic, such as isolation due to social distancing, are risk factors for mental health disorders [5]. For those whose insurance is tied to their employment, this also meant a loss of health insurance in the middle of the pandemic, at a time when they may have needed it most. Indeed, a rise has been observed in mental health disorders and feeling of anxiety, depression, boredom, and anger [5].

The LGBTQ+ community in the US has been disparately impacted by the COVID-19 pandemic. Relative to non-LGBTQ+ people, LGBTQ+ folx are more likely to work in highly affected industries, such as food service, healthcare, and retail, have higher rates of baseline poverty (22% vs 16%), and lower rates of health insurance [3, 6]. Since the pandemic began, 64% of LGBTQ+ households experienced employment loss compared to 45% of non-LGBTQ+ households, and 66% of LGBTQ+ households have had serious financial issues compared to 44% of non-LGBTQ+ households [7]. For example, in one COVID study, 11% of all men who have sex with men (MSM) participants reported losing employment, and four out of ten anticipated losing at least 30% of their income [8]. Another COVID study showed that a large proportion of MSM reported challenges buying food and paying rent in the midst of decreased work hours and increased supportive financial needs from family [9].

Studies on the effect of the COVID-19 pandemic on the mental health of LGTBQ+ individuals have found that LGBTQ+ people had high scores for perceived stress and depressive symptoms, higher than observed in community samples and vulnerable populations pre-pandemic [10–12]. LGBTQ+ people who reported higher rates of discrimination during the pandemic, including verbal harassment and exclusion from social events, scored higher on the perceived stress scale. Transgender Gender Non-Conforming and Nonbinary (TGNCNB) people and young LGBTQ+ folx were also at higher risk of showing signs of stress and depression [11]. Additional studies showed that many LGBTQ+ respondents who had slight/moderate disparities in mental health relative to the overall population pre-pandemic, transitioned to clinically diagnosable disorders during the pandemic [12].

LGBTQ+ folx were especially at higher risk of showing signs of stress and depression [11]. Many LGBTQ+ youth have been forced to spend more time with or return to unsupportive, homophobic and transphobic biological families during the pandemic due to schools moving to a remote model. 60% of LGBTQ+ college students reported frequent mental distress, anxiety, or depression, with higher rates among those with unsupportive families [13]. As mental health outcomes may be related to level of familial support, this may contribute to higher risk of poor mental health outcomes during the pandemic [14, 15]. At the other end of the age spectrum, LGBTQ elders are isolated by living alone and likely single, and are less likely to have immediate families to support them through the pandemic [3].

Much research has been conducted on the ways in which individual identifications such being LGBTQ+ or BIPOC influences health, as described above. For example, mental health concerns are particularly prevalent for historically marginalized groups such as LGBTQ+ folx and BIPOC folx; Black Americans have the highest COVID-19 mortality rate among all racial groups and therefore may be at higher risk of developing mental health concerns than other racial groups [5].

Applying an intersectionality framework, this study examines the ways in which, for example, the stressors related to being Black *and* LGBT contribute to one’s health, especially when one experiences racism in LGBT communities or homophobia in Black communities [16]. This is the first study to measure perceived stress during the beginning of the COVID pandemic, and how that stress was differentially impacting members of the LGBTQ+ community, as they encompass multiple historically marginalized identities.

## Materials and methods

This is a secondary analysis of an international study of non-medical impacts of COVID on individuals during the beginning of the COVID pandemic, April-May 2020. The original study was informed by the Critical Medical Ecology model [17]; recruited respondents from six geographical regions of Africa, Asia, Europe, Latin American and the Caribbean, North America, and Oceania; and was available in English, Spanish, Italian, and French. The online survey was advertised via Facebook, Instagram, and the Facebook Audience Network, plus the Amazon Mechanical Turk (‘mTURK’) online workforce [18, 19]. The original sample included 7411 participants from 173 countries; additional sampling information is available [20].

For these secondary analyses, data was restricted to US respondents. The exposure, gender identity and sexual orientation (SOGI), was determined via quantitative questions on gender and sexual orientation, plus review of open ended responses to the choice of “other” after each question. If “other gender” was described as transgender or nonbinary, then the transgender variable was changed to indicate “yes”. If the “other orientation” listed heterosexual, Christian, married widow, divorced, or normal, the sexual orientation was changed to “straight”. If “other orientation” listed pansexual, panqueer, or queer, then a new variable of queer was created and indicated as “yes”; these individuals were counted as members of the LGBTQ+ community. Data cleaning yielded 180 gays, 71 lesbians, 469 bisexuals, 33 transgender individuals, and 12 queer individuals. The LGBTQ+ community had 714 members while the LGBQ+ community had 700 members, recognizing that some transgender individuals are members of the LGBQ+ community while others are not. Respondents were asked a variety of questions about access to food and medical services, plus the validated Perceived Stress Scale (PSS), which was coded from a scale of 0 to 40, and recoded into 1–13 as low stress, 14–26 as moderate stress, and 27+ as high stress [21].

Descriptive univariate and bivariate analyses were conducted using chi-square tests for dichotomous variables and t-tests for categorical variables. Ordinal regression was conducted to determine the relationship between SOGI and PSS, controlling for variables that were significant in bivariate analyses and those determined *a priori* to belong in the model. To recognize the intersectional lives that participants lead [16, 22], interaction variables tested SOGI status with race, ethnicity, income, and dis/ability status, as well as each of these variables with each other (i.e. race*ethnicity, income*dis/ability). Sensitivity analyses were conducted by identifying SOGI status as LGBTQ+ or LGBQ+ and by dichotomizing PSS scores as low/medium vs. high and low vs. medium/high.

This study was performed in accordance with the ethical standards established by the 1964 Declaration of Helsinki and its later amendments. The University of Rochester’s Research Subjects Review Board determined that this study met federal and University criteria for exemption (STUDY00004825). All participants provided informed consent to engage in this research after a review of a detailed Information Sheet presented in English, French, Spanish or Italian at the beginning of the REDCap survey.

## Results

A total of 1,362 US participants were included in the analyses, including 72.3% (n = 986) straight/heterosexuals, 2.5% (n = 34) gays, 1.6% (n = 22) lesbians, 10.1% (n = 137) bisexuals, 1.0% (n = 13) transgender individuals, and <1% (n = 7) queers, yielding 14.2% (n = 193) LGBTQ+ community members and 14.0% (n = 191) LGBQ+ community members (Table 1).

**Table 1:** Demographic characteristics of sample of US-based LGBTQ+ community members in the first year of the COVID pandemic.

	LGBTQ+ community member n(%)	Not member of LGBTQ+ community n(%)	Total n
Demographics			
Race*			
White	114 (12.9)	772 (87.0)	886
Any Other Race	60 (27.0)	162 (73.0)	222
Ethnicity			
Hispanic or Latinx	138 (15.3)	766 (84.7)	904
Not Hispanic or Latinx	51 (20.2)	202 (89.8)	253
Income*			
Neither car nor home	28 (20.9)	106 (79.1)	134
Owns car or home	72 (21.62)	261 (78.4)	333
Owns car and home	86 (11.1)	691 (88.93)	777
Ability*			
Hearing/Non-deaf	77 (14.0)	472 (86.0)	549
Hard of Hearing, deaf, Deaf, DeafBlind	69 (31.4)	151 (68.6)	220
Age*			
18–24	20 (33.9)	39 (66.1)	59
25–34	89 (25.7)	258 (74.4)	347
35–44	33 (15.4)	181 (84.6)	214
45–54	20 (12.7)	137 (87.3)	157
55–64	19 (8.0)	219 (92.0)	238
65+	7 (4.0)	169 (96.0)	176
Education			
Did not complete high school	2 (14.3)	12 (85.7)	14
Completed secondary education, high school, or GED	13 (12.3)	93 (87.7)	106
Attended university/college and did not complete	20 (11.8)	149 (88.2)	109
Graduated from university/college	104 (17.9)	478 (82.1)	582
Degree beyond university/college (MA, PhD, MD, etc)	48 (16.7)	239 (83.3)	287
Gender*			
Male	102 (18.5)	449 (81.5)	551
Female	76 (12.3)	540 (87.7)	616
TGBCNB	13 (65)	7 (35)	20
Outcome			
Perceived stress scale*			
Low	20 (6.3)	297 (93.7)	317
Moderate	130 (18.1)	590 (81.9)	720
High	22 (16.9)	108 (83.1)	130

*statistically significant distribution between variables, p<0.05

TGNCNB = transgender, gender nonconforming, nonbinary

**Fig 1:**
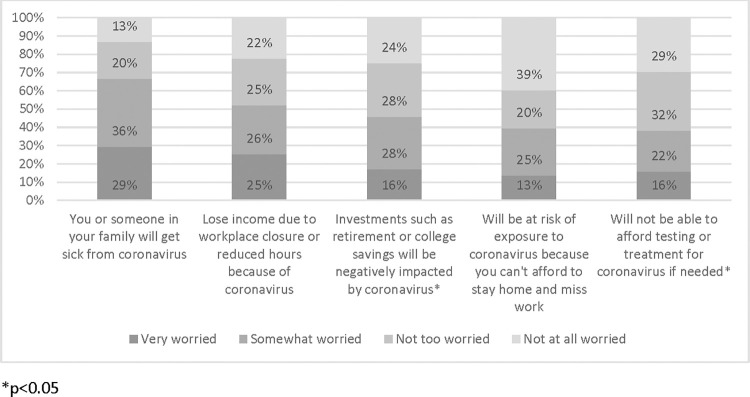
Worries of US-based LGBTQ+ respondents in the first year of the COVID pandemic.

LGBTQ+ community members were similar to their non-LGBTQ+ counterparts in several ways. Among LGBTQ+ community members, 80.0% felt they had enough information to protect themselves and their family from coronavirus; 31.4% felt that coronavirus-related worry or stress had a major negative impact on their mental health while 34.0% felt a minor mental health impact (Fig 1). Participants were asked how worried they were about someone in their family getting sick from coronavirus, losing income due to a workplace closure or reduced hours, and putting themselves at risk of exposure to coronavirus because they can’t afford to stay home and miss work, with statistically significant differences seen concerning investments and affording testing or treatment (p < .05).

**Fig 2:**
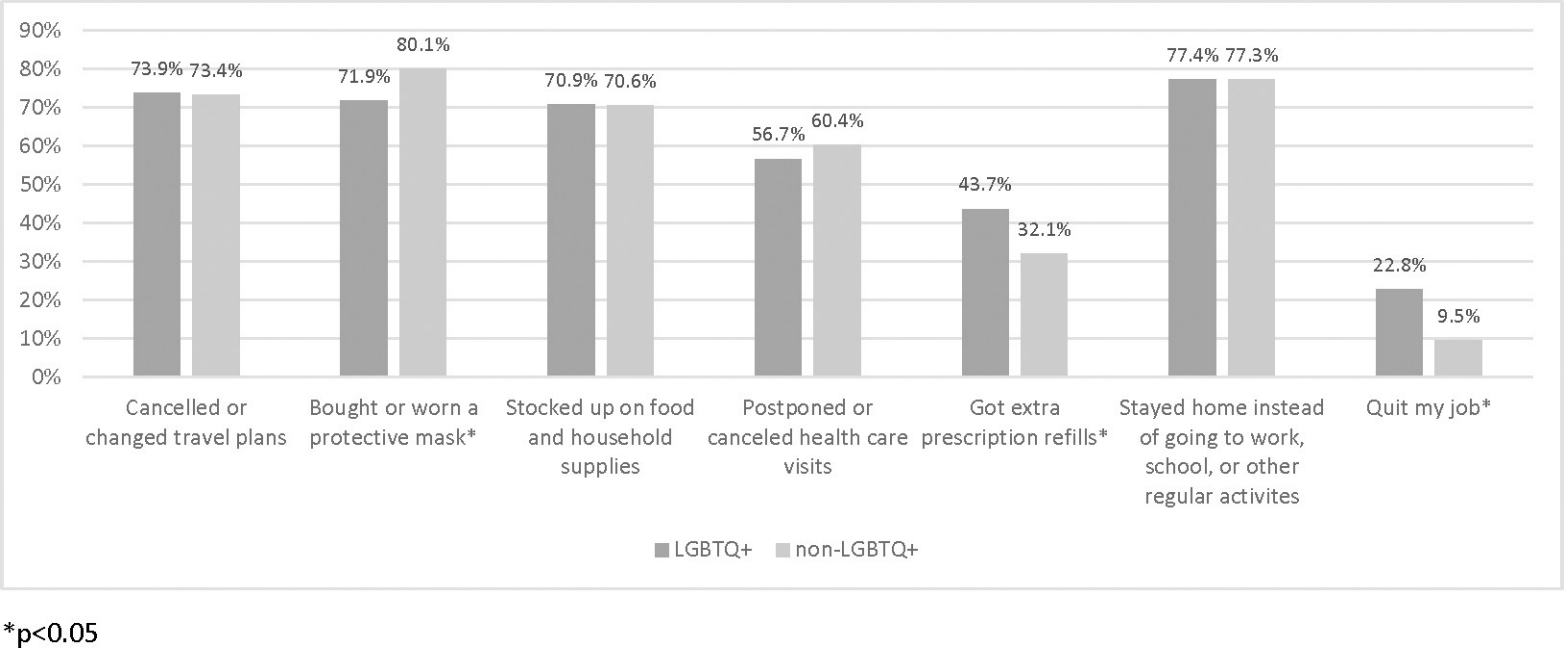
Responses of US-based LGBTQ+ community members on how COVID-19 impacted their activities.

LGBTQ+ community members were different from non-LGBTQ+ counterparts in several activities, including being less likely to buy or wear a protective mask, more likely to get extra prescription refills, and more likely to quit their jobs (p < .05, Fig 2).

**Fig 3:**
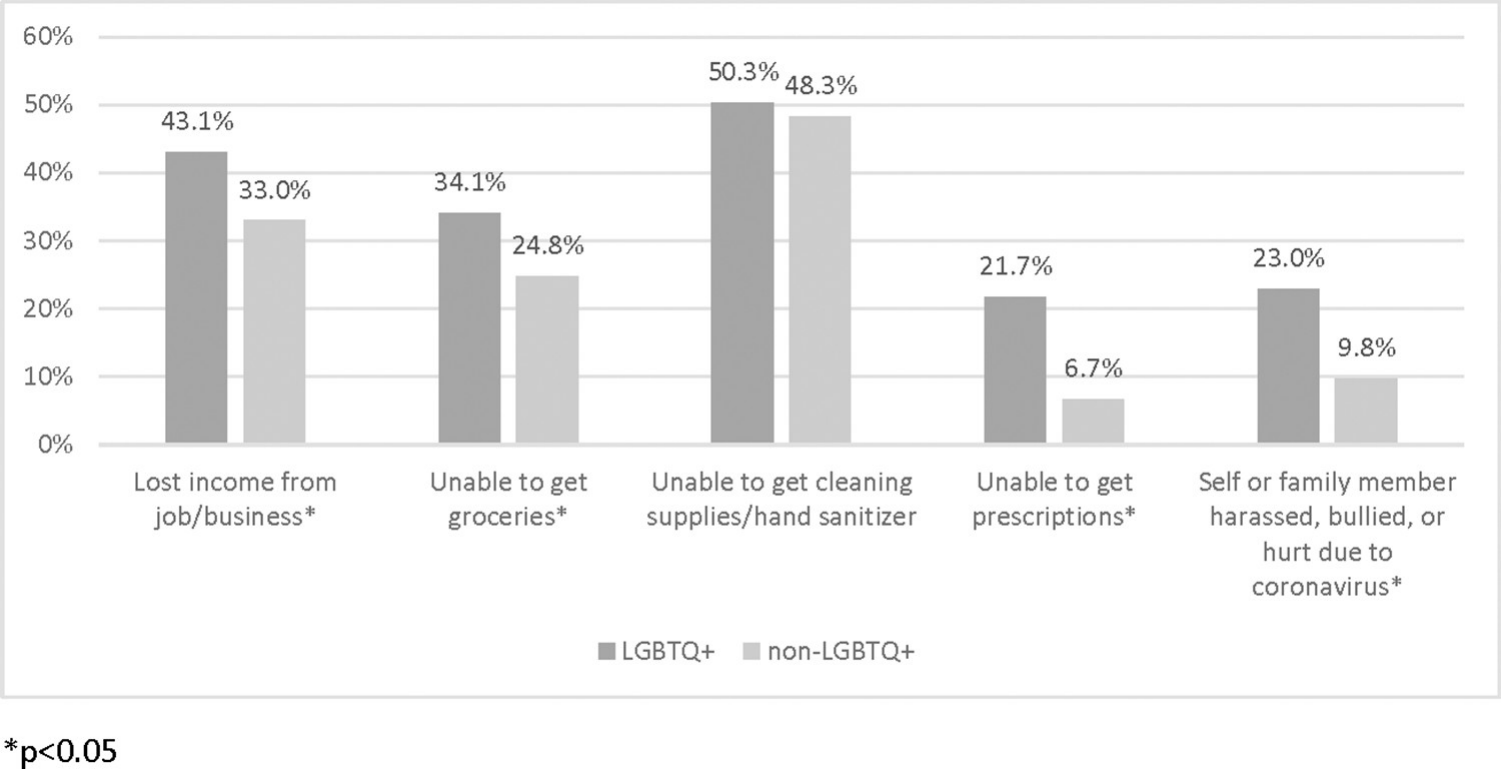
Experiences of US- based LGBTQ+ community members on access to resources in the first year of the COVID pandemic.

There were statistically significant differences in how coronavirus impacted the everyday experiences of LGBTQ+ community members versus non-LGBTQ+ folx, in terms of increased lost income, increased inability to get groceries or prescription medication, and increased reporting that they or their family member had been harassed, bullied, or hurt due to coronavirus (p < .05, Fig 3).

Ordinal and logistic regression was conducted using intersectionality as the theoretical framework for experience as an LGBTQ+ community member. Members of the LGBTQ+ community had 2.01 (95%CI 1.45–2.79) times the odds of having moderate compared to low PSS scores, and then high compared to moderate PSS scores, compared to non-LGBTQ+ community members. BIPOC LGBTQ+ community members had 2.09 (95%CI 1.21–3.58) times the odds of having moderate compared to low PSS scores, and then high compared to moderate PSS scores, compared to white non-LGBTQ+ community members. Adjusting for Hispanic ethnicity, income, age, and education, BIPOC LGBTQ+ identification was not significantly associated with higher PSS scores compared to white non-LGBTQ+ identification. However, adjusting for Hispanic ethnicity and income, BIPOC LGBTQ+ community members had 2.02 (95%CI 1.16–3.53) times the odds of having moderate compared to low PSS scores, and then high compared to moderate PSS scores, compared to white non-LGBTQ+ community members.

Deaf LGBTQ+ community members had 2.27 (95%CI 1.30–3.96) times the odds of having moderate compared to low PSS scores, and then high compared to moderate PSS scores, compared to hearing non-LGBTQ+ community members. Adjusting for Hispanic ethnicity, income, age, and education, deaf LGBTQ+ community members had 2.00 (95%CI 1.12–3.61) times the odds of having moderate compared to low PSS scores, and then high compared to moderate PSS scores, compared to hearing non-LGBTQ+ community members (S1 File).

## Discussion

The advent of COVID has added yet another stressor that impacts different historically marginalized identities- and diverse compositions of historically marginalized identities- in unique ways. Factors such as lower socioeconomic status, less preventative health education and intervention, less access to healthy food and adequate housing and more all play a part in almost all historically marginalized categories across the board experiencing higher rates of COVID morbidity and mortality than those who hold dominant identities such as white, cisgender, heterosexual, wealthy, able-bodied, hearing, and male. The intersectional analytical lens applied to this data indicates that the LGBTQ+ community reported twice the increased perceived stress than non-LGBTQ+ community members in the first year of the pandemic. This finding held true for BIPOC LGBTQ+ community members and for deaf LGBTQ+ community members. These results support the interdependency of experiences at the intersection of racialized and disabled LGBTQ+ community members, causing us to ask how we can support these marginalized communities in all the ways they need services and support to reduce stress levels.

While SOGI data was not collected for the majority of COVID-19 studies [23–25], these findings can be compared to the other intersectional experiences of the LGBTQ+ community, based on race, ethnicity, income levels, and disabilities measured separately. Compared to white people, Native Americans, Black/African Americans, and Latinxs are 1.0–1.6 times more likely to get COVID, 2.5–3.3 times more likely to be hospitalized for COVID, and 1.9–2.2 times more likely to die from COVID [26]. In addition, for every 1% increase in a county’s income inequality level, there was an associated 2% increase in COVID incidence and a 3% increase in COVID deaths. This very closely mimicked the statistics for every 1% increase in a county’s Black or Latino population [27]. Important and life-changing appointments and procedures which people need for non-COVID conditions have been stalled or cancelled altogether as hospital resources are overwhelmed. Telehealth can be especially difficult to navigate for the deaf, mute, or visually impaired, as well as those with learning disabilities/cognitive delays. People who require caregivers may not have access to them with the rules on physical isolation, and additionally be unable to travel independently to receive medical treatment as needed [28].

This study has several limitations. While data was collected about 1.5 years ago, it captures early COVID-19 stressors. There was a very small transgender population (n<20), preventing any subanalyses. While the deidentified nature of data may have increased transparency, it prevented follow-up. The implications of this study are impacted by the fact that very little public health COVID-19 data has collected SOGI information [23–25], limiting the ability to assess true COVID-19 impacts on the LGBTQ+ population. Lastly, an intersectional lens was applied post hoc to existing data, necessitating an additive analytical model instead of a preferential intersectional measurement and analyses [29].

Intersectionality shapes other pieces of how COVID-19 impacts the lives of LGBTQ+ community members. Unemployment and reduction of work hours has significantly increased for LBGTQ+ and BIPOC LGBTQ+ community members especially. As vaccines were not available at the time of data collection, we measured interest in obtaining vaccination when one was available. A recent study on vaccination uptake among the LGBTQ+ community shows mixed results. While transgender adults, including BIPOC transgender adults, are more likely to get vaccinated (53% and 47% respectively, versus 39% of all adults, Black LGBTQ+ and Latinx LGBTQ+ adults are less likely to get vaccinated (29% and 30% respectively, versus 42% LGBTQ+ adults [30]. As the public health community attempts to vaccinate ourselves out of the pandemic, it’s crucial to understand that vaccine hesitancy is impacted by: (1) historical racial discrimination of BIPOC communities by medicine, (2) lack of connection to primary care providers of whom to ask questions about efficacy and side effects, (3) income insecurity and lack of health insurance, and (4) involvement of government in vaccine development and testing [31–33]. While none of these ideas or concerns are new to the LGBTQ+ and/or historically marginalized communities, vaccination messages need to address the aforementioned legitimate concerns.

There are several implications for this study, including that it is important to identify and mitigate COVID-related stress and it is critical to interpret perceived stress through an intersectional lens, *especially for LGBTQ+ community members*.

## Conclusion

It is imperative for the public health community to identify ways to mitigate stress of BIPOC and deaf LGBTQ+ folx that addresses the intersectionality of their experience, including health insurance access and additional mental health concerns, within the context of unemployment or under employment. Persistent, damaging inequities due to COVID-19 that are largely preventable through deliberate attention, intervention, and policies intensify the challenges facing communities that have been traditionally excluded from public health and social care benefit.

## Supporting information

S1 File Technical appendix of regression coefficients. (DOCX)
