# Supplementary material for: Piling it on: Perceived stress and lack of access to resources among US-based LGBTQ+ community members during the COVID-19 pandemic
Source: PLoS One. 2022 Jul 8;17(7):e0271162. doi: 10.1371/journal.pone.0271162 (PMC9269365; doi:10.1371/journal.pone.0271162)
Supplement: S1 File — (DOCX) [file pone.0271162.s001.docx]

**S4. Technical Appendix of Regression Coefficients**

Regression 1. Unadjusted ordinal regression of LGBTQ+ identification as the independent variable and an ordinal dependent variable of Perceived Stress Scale with 1,167 observations and a pseudo R-squared value of 0.0085

| Variable | Coefficient | Standard Error | p-values | Confidence intervals |
| --- | --- | --- | --- | --- |
| LGBTQ+ member | .6982935 | .1666884 | 0.000 | .3715902, 1.024997 |

LGBTQ+=lesbian, gay, bisexual, transgender, and/or queer

Perceived Stress Scale coded as 0= low stress, 1= moderate stress, and 2= high stress

Regression 2. Interaction of race and LGBTQ+ identification as the independent variable and an ordinal dependent variable of Perceived Stress Scale with 988 observations and a pseudo R-squared value of 0.0139

| Variables | Coefficient | Standard Error | p-values | Confidence intervals |
| --- | --- | --- | --- | --- |
| LGBTQ+ x Race |  |  |  |  |
| non-LGBTQ+ and white | referent |  |  |  |
| non-LGBTQ+ and BIPOC | .2701629 | .1843985 | 0.143 | -.0912515, .6315773 |
| LGBTQ+ and white | .9116386 | .2232254 | 0.000 | .4741249, 1.349152 |
| LGBTQ+ and BIPOC | .7043549 | .2839262 | 0.013 | .1478697, 1.26084 |
| Non-Hispanic or Latinx Ethnicity | -.172275 | .1599424 | 0.281 | -.4857564, .1412064 |
| Income | -.1400903 | .2181851 | 0.521 | -.5677253 .2875447 |

Black, Indigenous People of Color (BIPOC)= American Indian, Asian, African American, Native Hawaiian, and/or Pacific Islander

LGBTQ+=lesbian, gay, bisexual, transgender, and/or queer

Perceived Stress Scale coded as 0= low stress, 1= moderate stress, and 2= high stress

Regression 3. Interaction of ability and LGBTQ+ identification as the independent variable and an ordinal dependent variable of Perceived Stress Scale with 668 observations and a pseudo R-squared value of 0.0350

| Variables | Coefficient | Standard Error | p-values | Confidence intervals |
| --- | --- | --- | --- | --- |
| LGBTQ+ x Ability |  |  |  |  |
| non-LGBTQ+ and Hearing/non-deaf | referent |  |  |  |
| non-LGBTQ+ and deaf | .2066441 | .2077876 | 0.320 | -.2006121, .6139003 |
| LGBTQ+ and Hearing/non-deaf | .6044361 | .2828795 | 0.033 | .0500025, 1.15887 |
| LGBTQ+ and deaf | .6936262 | .3012794 | 0.021 | .1031295, 1.284123 |
| Non-Hispanic or Latinx Ethnicity | .1126908 | .1899959 | 0.553 | -.2596944, .485076 |
| Income | -.1225258 | .2734385 | 0.654 | -.6584554, .4134038 |
| Age | -.2759939 | .0565837 | 0.000 | -.386896, -.1650918 |
| Education | -.0593676 | .0889522 | 0.505 | -.2337107, .1149755 |

LGBTQ+=lesbian, gay, bisexual, transgender, and/or queer

deaf= Hard of hearing, deaf, Deaf, and/or DeafBlind

Perceived Stress Scale coded as 0= low stress, 1= moderate stress, and 2= high stress
